# Supplementary material for: Tissue-specific mitochondrial pathway remodeling linked to longevity in honeybee queens
Source: PLoS One. 2026 Jan 28;21(1):e0341233. doi: 10.1371/journal.pone.0341233 (PMC12851464; doi:10.1371/journal.pone.0341233)
Supplement: S1 Fig — (DOCX) [file pone.0341233.s001.docx]

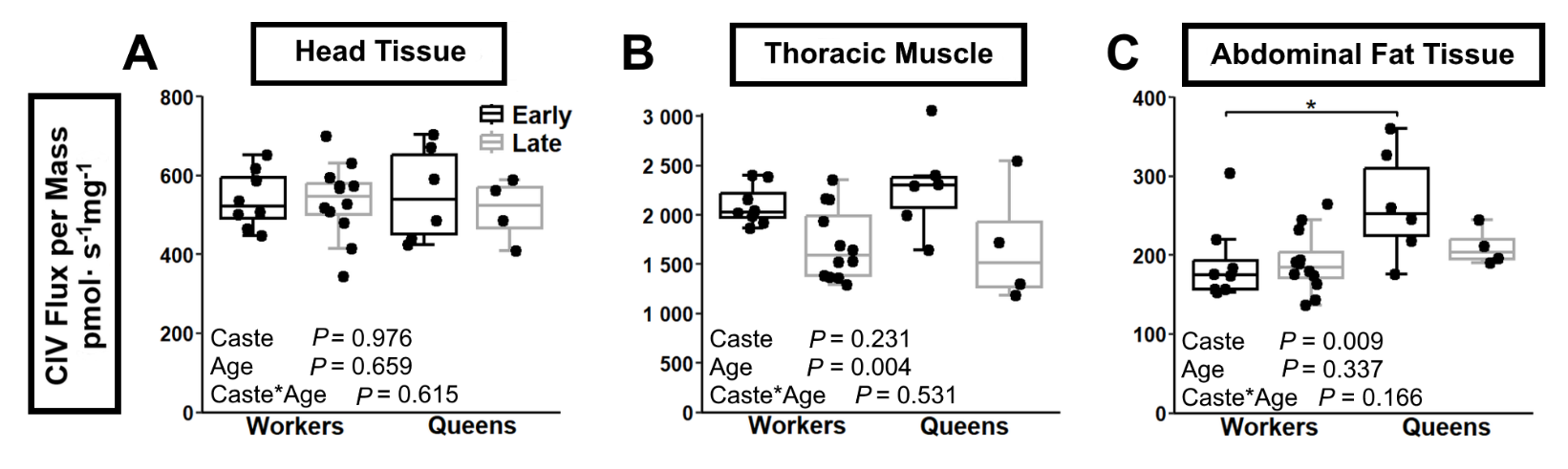


**Fig S1: Complex IV activity as an additional biomarker of mitochondrial content in worker and queen bees across different ages and body regions.** Complex IV activity is shown for the head tissue (A), thoracic muscle (B) and abdominal fat tissue (C) and normalized to tissue mass (per mg). Box plots display the minimum, 25th percentile, median, 75th percentile, and maximum values. Data represent workers at 1 week (early-life, *n* = 8) and 4 weeks (late-life, *n* = 12) and queen at 1 week (early-life, *n* = 6) and 109 weeks (late-life, *n* = 4). Two-way ANOVA *p*-values for the effects of bee caste (workers vs. queens), age, and their interaction are indicated in each panel. Significant differences between ages within a bee caste (shown below the boxes) and between bee castes at the same age (shown above the boxes) are denoted * (*P* ≤ 0.05), ** (*P* ≤ 0.01), and *** (*P* ≤ 0.001).
