# Supplementary material for: Tissue-specific mitochondrial pathway remodeling linked to longevity in honeybee queens
Source: PLoS One. 2026 Jan 28;21(1):e0341233. doi: 10.1371/journal.pone.0341233 (PMC12851464; doi:10.1371/journal.pone.0341233)
Supplement: S2 Fig — (DOCX) [file pone.0341233.s002.docx]

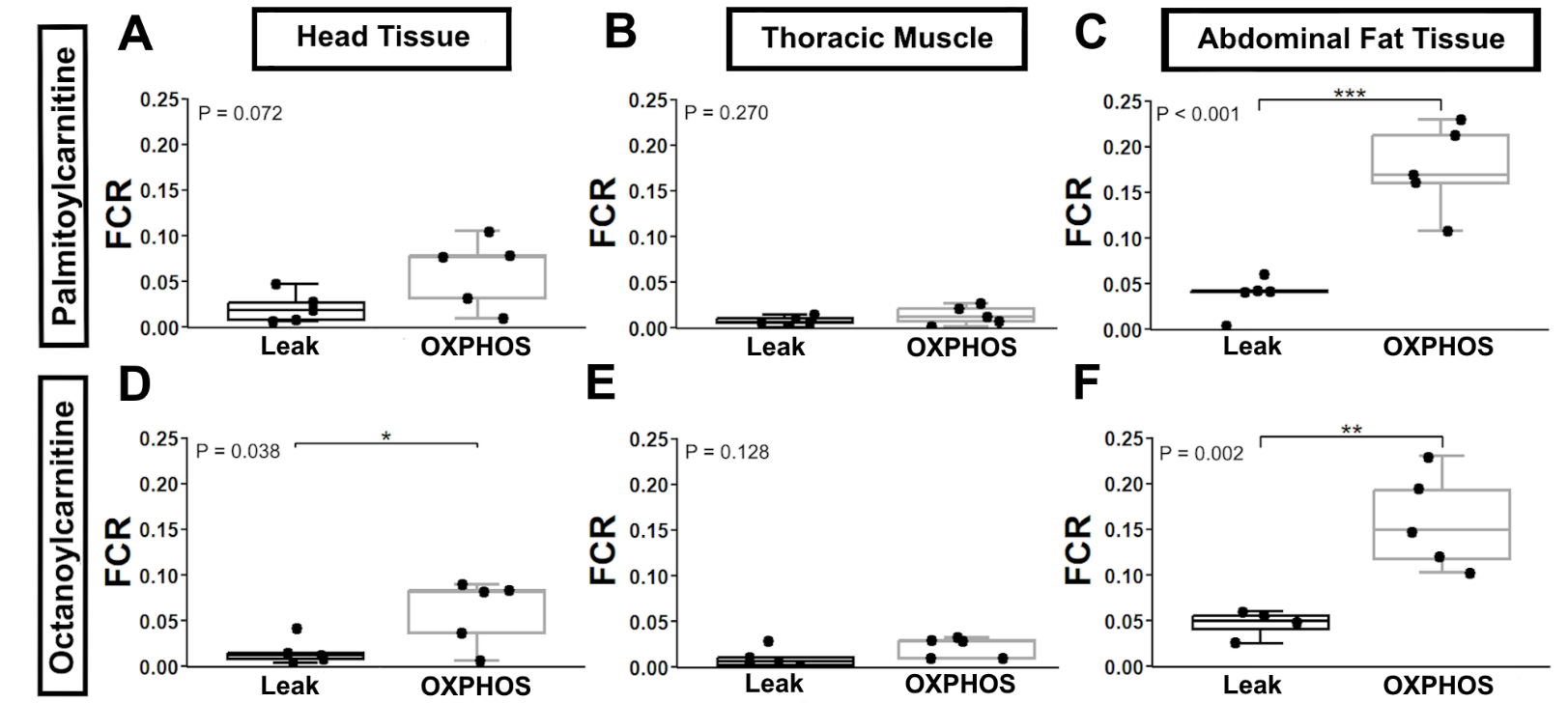


**Fig S2: Leak and OXPHOS capacity of fatty acid oxidation pathways in various tissues of worker bees.** Respiration is expressed as the flux control ratio (FCR), normalized to the maximal electron transport system (ETS) capacity after decoupling, based on the combined pathways flux. The data represented the leak state (in black) and the OXPHOS state (in grey) for the head tissue, the thoracic muscle and the abdominal fat tissue. Relative contribution of the fatty acid oxidation pathway (FAO-pathway) in the presence of palmitoylcarnitine (Panel A) or octanoylcarnitine (Panel B) relative to maximal flux (with combined FAO, NADH and Succinate pathways). Box plots display the minimum, 25th percentile, median, 75th percentile, and maximum values (n= 5). Two-way ANOVA *p*-values for the effect of ADP are indicated in each panel.
