# Supplementary figures and images for: Tissue-specific mitochondrial pathway remodeling linked to longevity in honeybee queens
Source: PLoS One. 2026 Jan 28;21(1):e0341233. doi: 10.1371/journal.pone.0341233 (PMC12851464; doi:10.1371/journal.pone.0341233)

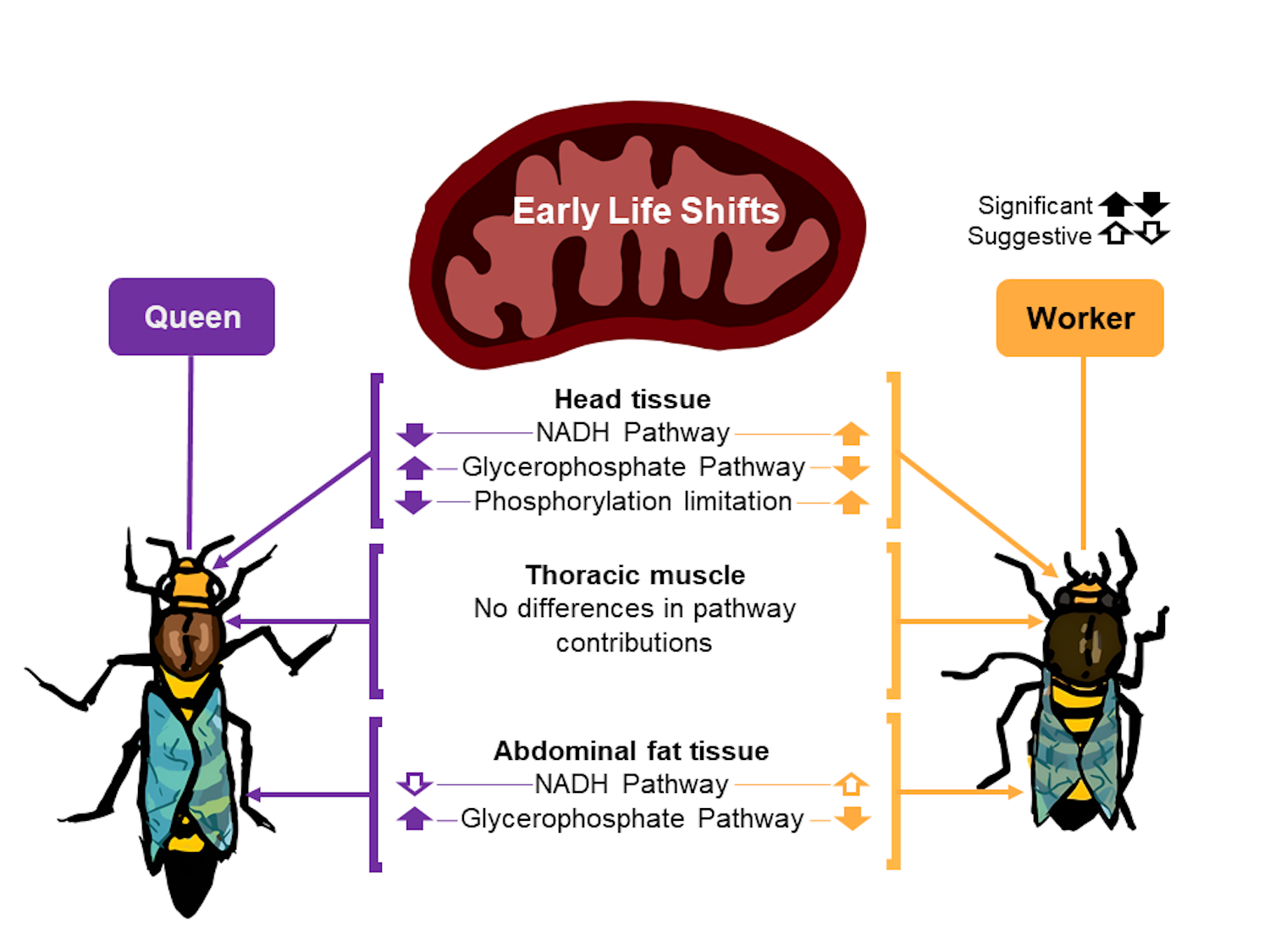

Supplement: S1 File — (TIF) [file pone.0341233.s003.tif]
