# Supplementary material for: Tissue-specific mitochondrial pathway remodeling linked to longevity in honeybee queens
Source: PLoS One. 2026 Jan 28;21(1):e0341233. doi: 10.1371/journal.pone.0341233 (PMC12851464; doi:10.1371/journal.pone.0341233)
Supplement: Attachment — Submitted filename: PLOS.pdf [file pone.0341233.s004.pdf]

The goal of PLoS ONE is to publish high quality studies irrespective of novelty in the field. I found that this study addresses an important and interesting (and novel) topic, but the quality of the study and presentation have significant limitations. I have substantial concerns about the methodology; as it was carried out, the approach limits the interpretability of the data. Some of these limitations are clear, and there are other places where the Methods are not complete enough to interpret the results. I focused my comments on the Introduction and Methods, because in my opinion, these substantial concerns would need to be addressed before it is worth addressing the Results and Discussion.

Overview of concerns

1) My biggest concern is the treatment and interpretation of the body compartments and tissues analyzed in this study, especially since the authors are attempting to compare across extremely different types of honey bees – queens and workers. The authors normalize their data to wet mass of the head, thorax, and eviscerated abdomen, and then compare these results across castes of bees (queens and workers) that differ substantially in the contents and sizes of these body compartments (most notably the head, which contains the brain, but also metabolically active glands). These compartments, as handled, contain tissues but also cuticle, which differs between the larger queen and smaller workers. The fact that the head masses of the queens and workers (all groups) did not differ in this study (Fig 1A) makes me suspicious that this measure of mass fails to capture real differences in the head tissue content that contribute to metabolic variation in this study. It is not prohibitively difficult to dissect the brain and other head tissues and the muscle from the thorax. The abdominal body fat is the cleanest single tissue preparation in this study, but here too, there is variable quantity of cuticle that may overwhelm any variation in tissue content itself. Also, you have omitted one of the most metabolically active organs of the queen, the ovaries, and it isn't clearly justified why (since you treat the thorax and head as if they are the same across queens and workers). It would be preferable to at least quantify some concrete measure of tissue, like protein content, which is a more standard and reliable way to assess variation in starting tissue quantity. However, the lack of consideration of the diversity of tissue types, especially in the heads of queens and workers, remains an issue even with these additional data.
2) Especially in the first section, the methods are not detailed enough here to make clear the relatedness, origin, and management of your various treatment groups; this makes the interpretation of the study very difficult. For example, nowhere in the methods do you describe how the bees were collected from the experimental hives.

This is critical because in honey bee behavior and biological age are tightly linked; in many cases, behavior supersedes absolute age in driving biological patterns. Did you collect behavior-matched bees, or were these different specializations (e.g., 7-day-old nurses versus 4-week-old foragers)? I found it similarly very difficult to parse the origins of the individuals used in this study, e.g., whether they were sisters. The handling of the mitochondrial samples was also very unclear. Please see more detailed comments below.

3) The Introduction is long, and many statements and terms are too general to be meaningful (e.g., "aging" and "mitochondrial metabolism"). It does not clearly build to the current study contribution. Please see my more detailed comments on the Introduction below.

Detailed comments:

1) In the future, please make sure you have continuous line numbering in your manuscript. Without line numbers it is very difficult to reference specific parts of the text.
2) More descriptive title? A little ambiguous term "shift"

Introduction:

1) First sentence – biological basis of "aging" sounds very general, and the statement about mitochondria that follows also seems overly general – for example, there are diverse theories about cellular, molecular, and tissue level changes associated with aging – some may have nothing to do with mitochondrial function directly, e.g., telomere shortening. I think it would be good to combine the first two paragraphs and then you can cut back on some of these overly general statements.
2) The third paragraph about social hymenoptera – omit "fine-tuned"' change phenotypic flexibility to plasticity; typo in "polyethism"; unclear what is meant by slow and then accelerated aging – please add more context. As with the earlier portions of the Introduction, it is problematic to use the word "aging" so generally. The only aspect or definition of "aging" that seems clear cut to the naïve reader is that time passes. Time can't pass more slowly or more quickly, so you need to qualify what you mean by aging here – you have some concept in mind but it is not explained; flight should be "flights"; clarify that queens' metabolic demands are distinct, but not necessarily lower – there are flight metabolic demands, but for many animals, the demands associated with yolking up eggs are substantial, and greater than any other activity; queen larvae "frequently"...omit frequently – they always receive larger amounts, workers are also fed frequently, but in small amounts; "These profound intraspecific differences..." – I am not sure one can say

these things are all “driven” by metabolic adaptations (again, omit finely tuned, the meaning is unclear). They are likely correlated with these changes. Mitochondrial function is both a cause and an effect, and the direction of the relationship is hard to disentangle.

3) “One limitation…” paragraph – again it seems like your terminology is overly general. “Mitochondrial metabolism” can mean a lot of things – metabolic phenotyping is often a combination of careful metabolomic analyses (with tracers), mitochondrial functional assays, and combinations thereof in well-controlled experiments. What you are describing here does not meet that level of criteria – throughout the Introduction, it is unclear what level you will address in this study and why that level of analysis is meaningful (I do not have an issue with the level you are addressing, it just is not clear why you have chosen it); I would not call this a “rare exception” in the context of mitochondrial metabolism. Please clarify earlier, and throughout, that the issue you are attempting to address is tissue-specific metabolism (and please qualify what “metabolism” means to you earlier in the Introduction); are “mitochondrial genes” nuclear genes that encode proteins that function in the mitochondria, or proteins encoded in the mitochondrial DNA, or both?; This is a study that measured body compartment-specific mitochondrial function in honey bees – the phrasing in this paragraph suggests you may be unaware of it

Cervoni, M.S., et al., Mitochondrial capacity, oxidative damage and hypoxia gene expression are associated with age-related division of labor in honey bee (Apis mellifera L.) workers. J Exp Biol, 2017. 220(Pt 21): p. 4035-4046.

4) “The objective…” paragraph – is it necessary to add the term “cross-sectional” (the meaning is not immediately clear) or could you just state that you are comparing queens and workers?; by “aging” I assume you mean comparing early and late in life, by “task specialization” I assume you mean queen versus worker, but by “longevity” I assume you mean, within each of these groups, individuals or genetic strains that show lifespan differences. Is this what you mean?; the “head” is not a tissue, as you clarify when you describe its contents (at least the brain). Please make sure you do not refer to the head as a tissue. Also, the glands of the head are very metabolically active, and differentially so for castes and worker specializations. I would assume they will contribute a lot to metabolic variation; “The functional measurement…” I think you mean different pathways that are linked to oxidative phosphorylation, as I think most would consider oxidative phosphorylation a pathway in and of itself; throughout this paragraph I do not see a clear justification for why you are focusing on this component of mitochondrial function. I think if you

reduce the generalities in the earlier parts of the Introduction, this will be easier to pull together.

Methods:

1) 2.1 – the methods are not detailed enough here to make clear the relatedness, origin, and management of your various treatment groups. Also, there are no citations here in this paragraph even though these methods are extensively used by a lot of groups; Were young queens mated or unmated, or variably so? Did you keep track of mating flights?; I think you mean "plastic tubs", not "tubes"; You say that "Late-life queens…the same origin colony as workers and early-life queens." This sounds like you are basically using sisters for all individuals in your treatment groups (queens and workers), but the details here are scant. For example, did you generate 1-day-old queens simply from emerging brood frames? I would assume you did something else to generate these queens from the source colonies. Please differentiate the source colony (which is important for relatedness and genotype) from the experimental colony, which sounds like it was simply used to house individuals once marked. How many source and experimental hives did you use? Stress levels and worker and queen physiology can change substantially across the season. What was the timeframe for the experiment exactly, and did you assess whether the date of introduction to the experimental colony influenced your results? Late-life queens were 109 weeks old, so those were not generated at the same time as the early queens and workers, correct? How were these queens managed exactly over this long timeframe? Sounds like they came from distinct colonies? N=4 is a small number. I know it is hard to track queens this long, and this is probably part of the low sample size.
2) "Immediately after homogenate preparation…" – what does this timeframe look like, and what is the approximate range of time from anesthetization to analysis for various individuals and tissues? Mitochondrial function varies substantially as a function of the tissue preparation and extraction protocols. Were all individuals assessed on the same day and in the same oroboros run? How were the samples handled to control for effects of time and handling?
3) It seems a bit problematic to compare across "tissues" on the basis of mass, since you weighed full body compartments (the head, the thorax, the abdomen with the gut removed). There is going to be a lot of variation in cuticular mass that is unaccounted for. Did you use a protein assay, e.g., to quantify total protein? That would be a better base of comparison. At the very least, throughout you should not say "per mg tissue" – you are not assessing single tissues (especially in the head), and there is the issue of the cuticle as well.
