# Supplementary material for: Tissue-specific mitochondrial pathway remodeling linked to longevity in honeybee queens
Source: PLoS One. 2026 Jan 28;21(1):e0341233. doi: 10.1371/journal.pone.0341233 (PMC12851464; doi:10.1371/journal.pone.0341233)
Supplement: Attachment — Submitted filename: 2025-12-08-Response to reviewers.pdf [file pone.0341233.s005.pdf]

Note: The line numbers in our point-by-point description correspond to the Revised manuscript with track changes.

Reviewer 1

This study presents high-resolution respirometry results comparing young vs. old queen and worker honey bees, with the implicit aim of gaining insights into the possible role of mitochondrial function (OXPHOS pathways) in shaping the immense lifespan difference between the castes of social insects. After comparing body compartment size and of citrate synthase activity as a proxy of functional mitochondrial units, the experiments were performed on homogenates of head (mainly brain), thorax (mainly flight muscle) and abdominal (mainly fat body) tissues using an Oroboros system to measure the age-, caste-, and tissue-specific rates of mitochondrial respiration. The experiments were carefully performed and the protocols are described in sufficient detail to serve as guidance for follow-up studies building on this approach and the obtained results. The detailed methods description is also of importance readers not familiar with this methodology, so that they can capture and understand the results and the respective conclusions.
This said, I only have a view comments and questions to this otherwise well-prepared manuscript.

Thank you for your positive and constructive comments. We have carefully considered each point and addressed them in detail below.

1) A critical issue in presenting comparative HRR data is the normalization step. As far as I understand, the flux control ratios (FCRs) were calculated by normalization against the non-mitochondrial residual oxygen consumption (ROX). This, however, does not take into account the changes in functional mitochondrial units along the bees' lifespan and tissue type, as shown in Fig 1, where this data is reported as citrate synthase activity, and where differences are clearly apparent for the head and abdomen. I certainly would expect that this factor can affect the interpretation of the results.

The flux control ratios (FCRs) provide a quantitative "fingerprint" of mitochondrial coupling and respiratory control by expressing each respiratory state relative to a common internal reference. In sequential titration protocols, FCRs enable internal normalization of oxygen flux, eliminating the need to correct for mitochondrial content (although we also quantified it here using citrate synthase activity). Each FCR represents the ratio of oxygen flux in a given mitochondrial respiratory state (Yi) to that in a defined reference state (Z), typically the maximal electron transfer system (ETS) capacity. In this study, the reference state corresponds to the uncoupled ETS condition, where NADH-, succinate-, glycerophosphate-, and fatty acid oxidation–linked pathways are all active. In the earlier version of the manuscript, FCRs were defined in the Methods section and figure legends (Figs 2–4). To enhance clarity, we have now added the explicit formula and expanded the explanation to illustrate more clearly how this ratio is calculated and interpreted (Lines 236-241).

''Alternatively, respiration was expressed as the flux control ratio [FCR; (39, 44)], defined as the respiration of a specific pathway or complex (Yi) normalized to the rate Z, i.e., the maximal ETS capacity (NSGp- or FAONSGp-pathway).

$$FCRi = \frac{Yi}{Z}$$

FCRs, by comparing oxygen flux in different respiratory states to a common reference state, allow comparisons that are independent of mitochondrial content or assay conditions.''

Furthermore, to better illustrate how each respiratory step contributes to the calculation of its corresponding FCR, we have added a representative trace of the Substrate–Uncoupler–Inhibitor Titration protocol in the Methods section (Fig 1; Legends on Lines 213-223), allowing readers to visualize the sequential titrations, the identification of each respiratory state (Yi), and the point at which the maximal ETS reference state (Z) is reached.

2) The authors compared their results to those of two prior studies that used a similar approach, and they explain why their results differ from those of these prior studies. While I agree with the argument for the first study (Menai et al.), the second study (Cervoni et al.) was criticized for having been performed in intact tissue and not in a homogenate. This, however, relates to the question whether a study examining the cellular physiology should be performed on intact cells in a tissue context, or whether a homogenate with mitochondria separated from the cellular context would be better representation. Both have drawbacks, on the one hand less control over the accessibility of the added compounds to the (unpermeabilized) mitochondria, while on the other, the cytoplasmic context is lost.

A solid tissue has multiple cell layers embedded within extracellular matrices that create a significant diffusion barrier for (1) oxygen[1-3], (2) ADP and (3) respiratory substrates.[4] Even when these molecules reach the plasma membrane, they cannot freely cross it without specific transporters or membrane permeabilization, since ADP and most mitochondrial substrates (e.g., malate, succinate) are highly polar or charged and therefore impermeant to intact cell membranes without tightly regulated transport mechanisms. Consequently, diffusion into deeper cell layers is severely restricted. When an intact tissue chunk is placed in a respirometry chamber, only the outermost cell layers receive adequate oxygen and substrate supply. The tissue core rapidly becomes hypoxic or even anoxic, and most mitochondria within the inner cells never encounter the added substrates or ADP required to sustain oxidative phosphorylation. As a result, the oxygen consumption recorded reflects mainly a non-uniform, diffusion-limited respiration of the outer tissue layers, rather than the intrinsic mitochondrial capacity of the entire tissue.

For these reasons, mitochondrial respiration assays are routinely performed on isolated mitochondria, intact detached cells (or a single layer of attached cells), permeabilized cells or tissue fibers, or homogenates,[5] all of which ensure that mitochondria have unrestricted access to substrates, ADP, and oxygen, enabling accurate assessment of their functional capacity.

The manuscript was revised as follows to provide a clearer explanation:
'In Cervoni, Cardoso-Júnior (34), the minimal increase in respiration following ADP addition likely reflects limited restricted mitochondrial access due to tissue neither homogenized nor permeabilized. Because solid tissues contain multiple cell layers and extracellular matrices that limit diffusion of oxygen, ADP, and metabolic substrates, mitochondrial respiration is typically assessed in isolated mitochondria, permeabilized preparations, or homogenates (60) where mitochondria have unrestricted access to substrates, ADP, and oxygen.'' (Lines 541-545)

References cited above:

1. Pias SC. How does oxygen diffuse from capillaries to tissue mitochondria? Barriers and pathways. The Journal of physiology. 2021;599:1769-1782.
2. Scandurra FM and Gnaiger E. Cell respiration under hypoxia: facts and artefacts in mitochondrial oxygen kinetics. Advances in experimental medicine and biology. 2010;662:7-25.
3. Place TL, Domann FE and Case AJ. Limitations of oxygen delivery to cells in culture: An underappreciated problem in basic and translational research. Free radical biology & medicine. 2017;113:311-322.
4. Pajor AM. Sodium-coupled dicarboxylate and citrate transporters from the SLC13 family. Pflugers Archiv : European journal of physiology. 2014;466:119-30.
5. Doerrier C, Garcia-Souza LF, Krumschnabel G, Wohlfarter Y, Mészáros AT and Gnaiger E. High-Resolution FluoRespirometry and OXPHOS protocols for human cells, permeabilized fibers from small biopsies of muscle, and isolated mitochondria. Methods Mol Biol. 2018;1782:31-70.

3) One issue that is of relevance in bioenergetics and metabolism is the type of diet consumed by the experimental animals. In the case of honey bees, there is a major difference in the diet consumed by young workers (high protein/lipid) compared to old workers (essentially only carbohydrate) and to queens, where both

young and old queens receive royal jelly (high protein/lipid) from the workers. Adding a sentence or two on this would enrich the discussion.

We thank the reviewer for this valuable suggestion. We agree that caste- and age-specific diets are an important aspect of honeybee physiology and directly relevant to bioenergetic differences. In response, we have expanded the Discussion to include a dedicated section on the impact of dietary composition (Lines 568–574). The new text reads:

"These pathway differences are likely influenced by caste-specific adult diets. Workers rely predominantly on carbohydrate-rich foods (nectar and honey) to fuel their high activity levels, whereas queens are continuously fed royal jelly, a secretion rich in proteins and lipids provided by nurse workers (30). This nutrient supply favors the use of non-carbohydrate substrates in queens and may promote greater engagement of alternative mitochondrial pathways. Such metabolic flexibility could support queens' sustained reproductive output and contribute to their remarkable lifespan."

We believe that this addition strengthens the discussion by linking mitochondrial pathway use with well-established nutritional differences among castes and worker age groups.

4) The legends for Figures 2-4 are very similar, showing the datasets separately for head, thorax, and abdominal tissues. Hence, for Figures 3 and 4 the legend text could be condensed.

We appreciate the reviewer's suggestion to streamline the figure legends. In response, we have revised the legends for Figs 3 (Lines 333-353), 4 (Lines 362-377) and 5 (Lines 379-389) to reduce redundancy while ensuring that each figure remains fully interpretable on its own. Methodological details and pathway definitions that were repeated across legends have now been condensed, with cross-references to Fig 3 where appropriate. This approach minimizes repetition while preserving clarity and scientific completeness.

5) Unfortunately I could not access the data deposited in DRYAD, but I trust that they will be made available upon publication.

Thank you for noting this. Yes, the data will be made publicly available upon acceptance of the manuscript, in accordance with the journal's data-sharing policy. We have already prepared the dataset and uploaded it to DRYAD, and it will be fully accessible once the paper is formally accepted.

Reviewer 2

The goal of PLoS ONE is to publish high quality studies irrespective of novelty in the field. I found that this study addresses an important and interesting (and novel) topic, but the quality of the study and presentation have significant limitations. I have substantial concerns about the methodology; as it was carried out, the approach limits the interpretability of the data. Some of these limitations are clear, and there are other places where the Methods are not complete enough to interpret the results. I focused my comments on the Introduction and Methods, because in my opinion, these substantial concerns would need to be addressed before it is worth addressing the Results and Discussion. I provide details about my concerns in the attached document.

Thank you for your feedback. We have done our best to address all comments and clarify the manuscript accordingly.

Overview of concerns
1) My biggest concern is the treatment and interpretation of the body compartments and tissues analyzed in this study, especially since the authors are attempting to compare across extremely different types of honey bees – queens and workers. The authors normalize their data to wet mass of the head, thorax, and eviscerated abdomen, and then compare these results across castes of bees (queens and workers) that differ substantially in

the contents and sizes of these body compartments (most notably the head, which contains the brain, but also metabolically active glands). These compartments, as handled, contain tissues but also cuticle, which differs between the larger queen and smaller workers. The fact that the head masses of the queens and workers (all groups) did not differ in this study (Fig 1A) makes me suspicious that this measure of mass fails to capture real differences in the head tissue content that contribute to metabolic variation in this study. It is not prohibitively difficult to dissect the brain and other head tissues and the muscle from the thorax. The abdominal body fat is the cleanest single tissue preparation in this study, but here too, there is variable quantity of cuticle that may overwhelm any variation in tissue content itself. Also, you have omitted one of the most metabolically active organs of the queen, the ovaries, and it isn't clearly justified why (since you treat the thorax and head as if they are the same across queens and workers). It would be preferable to at least quantify some concrete measure of tissue, like protein content, which is a more standard and reliable way to assess variation in starting tissue quantity. However, the lack of consideration of the diversity of tissue types, especially in the heads of queens and workers, remains an issue even with these additional data.

We appreciate the reviewer's thoughtful comments on tissue selection and normalization. Below, we clarify our rationale and address each point in turn.

We would like to emphasize that none of the conclusions in this study are based on the absolute mass of the head between queens and workers. Tissue wet mass was used solely to estimate total tissue quantity for normalization of mitochondrial content, not as a biological endpoint. Although the head masses did not differ significantly between castes in our samples (Fig 1A), the values were measured with a high-precision microbalance, and there is no published reference, to our knowledge, providing separate head-mass comparisons between queens and workers for validation. Importantly, the thorax and abdominal measurements in our dataset clearly captured the expected caste-specific differences in size, supporting the accuracy of our dissections and measurements.

We recognize that these compartments contain both cuticle and soft tissues, and that cuticle mass may differ between castes. However, our functional assays measure mitochondrial respiration and enzymatic activities of citrate synthase and complex IV, and maximal electron transfer system [ETS] flux, which are inherently restricted to the mitochondrial fraction and therefore unaffected by the non-metabolic components of cuticle. The normalization to tissue wet mass was used to express the total mitochondrial yield per unit of tissue, while all subsequent pathway-specific interpretations were derived from flux control ratios (FCRs), which are independent of tissue mass or mitochondrial content. As noted above (in the answers to reviewer 1), the FCRs provide a quantitative "fingerprint" of mitochondrial coupling and control by expressing each respiratory state relative to a common internal reference. In our sequential titration (SUIT) protocols, FCRs enable internal normalization of oxygen flux across samples, removing the need to correct for mitochondrial abundance. This analytical framework ensures that our comparisons reflect intrinsic differences in mitochondrial function rather than variation in total tissue or mitochondrial quantity. We have clarified this rationale in the revised text.

We agree that the ovaries are metabolically active organs in queens. However, they were intentionally excluded from this study because our objective was to compare homologous tissues, those shared by both queens and workers, to identify caste-specific metabolic patterns across equivalent body compartments (head, thorax, and abdominal fat body). Including a queen-specific organ would have precluded meaningful cross-caste comparisons.

2) Especially in the first section, the methods are not detailed enough here to make clear the relatedness, origin, and management of your various treatment groups; this makes the interpretation of the study very difficult. For example, nowhere in the methods do you describe how the bees were collected from the experimental hives. This is critical because in honey bee behavior and biological age are tightly linked; in many cases, behavior supersedes absolute age in driving biological patterns. Did you collect behavior-matched bees, or were these different specializations (e.g., 7- day-old nurses versus 4-week-old foragers)? I found it similarly very difficult

to parse the origins of the individuals used in this study, e.g., whether they were sisters. The handling of the mitochondrial samples was also very unclear. Please see more detailed comments below.

We have added additional information to the first paragraph of the Methods section (Lines 141–170) to address the reviewer's concerns. The revised text more clearly describes the relatedness, origin, management, and collection of the experimental groups. Specifically, we now specify that samples were collected at random from frames within hives originating from four different colonies, and subsequently placed into four separate host colonies. This clarification also addresses the comment regarding "behavior-matched" workers: although the workers were not explicitly matched for behavioral role, they were age-matched (from the same paint-marked cohort), and sampling was restricted to in-hive workers to avoid confounding age with behavioral status.

Regarding genetic similarity, we acknowledge the ongoing discussion in the literature about whether sampling from a single colony (to minimize genetic variation) or from multiple colonies (to increase generality across genotypes) is more appropriate. In the revised manuscript, we now clearly indicate the number of source colonies and note that all sampled bees originated from unselected commercial stock, representing a mixture of European lineages (Harpur et al. 2016, Insectes Sociaux 62(4): 479–489).

We hope that these additions provide sufficient detail to fully address the reviewer's comments.

3) The Introduction is long, and many statements and terms are too general to be meaningful (e.g., "aging" and "mitochondrial metabolism"). It does not clearly build to the current study contribution. Please see my more detailed comments on the introduction below.

We have carefully revised the Introduction to improve focus, clarity, and logical progression toward the objectives of the study. Specifically, we streamlined general background sections on aging and mitochondrial metabolism, reduced redundancy, and refined terminology to make key concepts more precise and directly relevant to our research question. We also strengthened the final paragraphs to better articulate the specific knowledge gap addressed by this study and how our comparative approach in queens and workers contributes to understanding mitochondrial reprogramming and lifespan plasticity.

Each of the reviewer's detailed comments on the Introduction has been addressed point by point below.

Introduction below.
Detailed comments:
1) In the future, please make sure you have continuous line numbering in your manuscript. Without line numbers it is very difficult to reference specific parts of the text.

Line numbers have now been added throughout the manuscript. We apologize for the inconvenience this omission may have caused and appreciate your patience.

2) More descriptive title? A little ambiguous term "shift"

We thank the reviewer for this helpful suggestion. In response, we have revised the title to: "Tissue-Specific Remodeling of Mitochondrial Pathways Associated with Longevity in Honeybee Queens."
The term "remodeling" was chosen to replace "shift" because it more accurately reflects the coordinated and functional reorganization of mitochondrial metabolism observed across tissues, rather than a simple directional change. This wording also better conveys the mechanistic nature of our findings and clarifies the link between mitochondrial pathway reprogramming and caste-specific longevity in honeybees.

Introduction:
1) First sentence – biological basis of "aging" sounds very general, and the statement about mitochondria that follows also seems overly general – for example, there are diverse theories about cellular, molecular, and tissue

level changes associated with aging – some may have nothing to do with mitochondrial function directly, e.g., telomere shortening. I think it would be good to combine the first two paragraphs and then you can cut back on some of these overly general statements.

In response to the reviewer's comments, we have revised the opening section of the introduction (Lines 40–60) to more fully acknowledge the broad range of aging mechanisms proposed across taxa, including genomic stability, proteostasis, and nutrient-sensing pathways, in addition to mitochondrial processes. We also added a statement about telomere shortening, noting that this mechanism is not universal. For example, many invertebrates—including *Apis mellifera*—maintain active telomerase and stable telomeres throughout life (Korandová et al., 2016, Chromosoma 125: 405–411). We have also clarified the definition of aging at the beginning of the introduction and specified that our focus is on physiological aging.

The revised text now better reflects that, although aging encompasses multiple interconnected biological processes, many ultimately converge on mitochondria as central integrators of metabolic regulation and cellular homeostasis. This provides a clearer and more coherent conceptual transition to the present study's focus on mitochondrial remodeling and longevity in honeybee queens.

2) The third paragraph about social hymenoptera – omit "fine-tuned"' change phenotypic flexibility to plasticity; typo in "polyethism"; unclear what is meant by slow and then accelerated aging – please add more context. As with the earlier portions of the Introduction, it is problematic to use the word "aging" so generally. The only aspect or definition of "aging" that seems clear cut to the naïve reader is that time passes. Time can't pass more slowly or more quickly, so you need to qualify what you mean by aging here – you have some concept in mind but it is not explained; flight should be "flights"; clarify that queens' metabolic demands are distinct, but not necessarily lower – there are flight metabolic demands, but for many animals, the demands associated with yolking up eggs are substantial, and greater than any other activity; queen larvae "frequently"…omit frequently – they always receive larger amounts, workers are also fed frequently, but in small amounts; "These profound intraspecific diLerences…" – I am not sure one can say these things are all "driven" by metabolic adaptations (again, omit finely tuned, the meaning is unclear). They are likely correlated with these changes. Mitochondrial function is both a cause and an eLect, and the direction of the relationship is hard to disentangle.

We have provided a fully revised version of the paragraph that directly addresses every reviewer comment, corrects terminology, clarifies the "aging" issue, removes ambiguous wording, and adds precise context. More specifically, the following changes have been made:

1. We removed the term fine-tuned and replaced phenotypic flexibility with phenotypic plasticity in the revised paragraph (Lines 62-63).
2. We corrected the spelling to polyethism (Line 67).
3. Comments: "Unclear what is meant by slow and then accelerated aging'' and "It is problematic to use the word 'aging' so generally; qualify what is meant".
    a. Answer: We have clarified this section by explaining that nurse bees experience lower extrinsic mortality and slower physiological decline, whereas foragers experience accelerated physiological decline due to the demands and risks of foraging. This avoids using the term "aging" in an ambiguous way, and when used, it was specified as physiological aging, which refers to the progressive decline in biological function within cells, tissues, and organ systems as an organism ages, independent of chronological time (Lines 66-70). We have also added a definition of the term physiological aging in the first sentence of the introduction (Lines 40-41). In the revised text, we also removed generalized references to "aging" and replaced them with more specific processes, including physiological decline (Line 62-88). This addresses the ambiguity noted by the reviewer.
4. Comment: "Flight should be 'flights".
    a. Answer: The term was corrected to mating flights (Line 71).
5. Comment: "Clarify that queens' metabolic demands are distinct, but not necessarily lower."

    a. Answer: We revised this section to emphasize that queens' post-mating role involves substantial metabolic demands associated with continuous egg production, which for many animals can exceed other activities. This removes any implication that their metabolic rate is lower. (Lines 72-75)
6. Comment: "Queen larvae 'frequently'… omit 'frequently'."
    a. Answer: We removed the word frequently and clarified that queen-destined larvae consistently receive large amounts of royal jelly. (Line 76)
7. Comment: "These differences cannot necessarily be described as 'driven' by metabolic adaptations; mitochondrial function is both a cause and an effect."
    a. Answer: We revised the causal language throughout. The paragraph now states that these traits are associated with or correlated with differences in metabolic profiles, avoiding strong causal claims. (Lines 80-81)
8. Comment: "Need to clarify the uncertainty about whether metabolic profiles are set early in life or shaped in adulthood."
    a. Answer: We clarify the sentence specifying that it remains unknown whether caste-specific mitochondrial metabolic profiles are established during larval development, shaped by adult behavior and physiology, or influenced by both. (Lines 83-85)
9. Comment "Overall wording about metabolic differences should be less deterministic."
    a. Answer: We softened the statements to avoid deterministic interpretations and emphasized the need for further research to disentangle cause and effect. (Lines 83-88)

3) "One limitation…" paragraph – again it seems like your terminology is overly general. "Mitochondrial metabolism" can mean a lot of things – metabolic phenotyping is often a combination of careful metabolomic analyses (with tracers), mitochondrial functional assays, and combinations thereof in well-controlled experiments. What you are describing here does not meet that level of criteria – throughout the Introduction, it is unclear what level you will address in this study and why that level of analysis is meaningful (I do not have an issue with the level you are addressing, it just is not clear why you have chosen it); I would not call this a "rare exception" in the context of mitochondrial metabolism. Please clarify earlier, and throughout, that the issue you are attempting to address is tissue-specific metabolism (and please qualify what "metabolism" means to you earlier in the Introduction); are "mitochondrial genes" nuclear genes that encode proteins that function in the mitochondria, or proteins encoded in the mitochondrial DNA, or both?; This is a study that measured body compartment-specific mitochondrial function in honey bees – the phrasing in this paragraph suggests you may be unaware of it Cervoni, M.S., et al., Mitochondrial capacity, oxidative damage and hypoxia gene expression are associated with age-related division of labor in honey bee (Apis mellifera L.) workers. J Exp Biol, 2017. 220(Pt 21): p. 4035-4046.

We are providing a fully revised paragraph that addresses every reviewer comment with clear definitions, accurate terminology, and improved framing. The following specific changes have been made (Lines 90-108):

- Comments: "Your terminology is overly general. 'Mitochondrial metabolism' can mean many things… Please clarify earlier, and throughout, what level of metabolism you address and why it is meaningful." "What you are describing does not meet the criteria of full metabolic phenotyping… I do not have an issue with the level you are addressing; it's just not clear why you chose it."
    - Response: We agree that the term "mitochondrial metabolism" is overly broad and may be interpreted in multiple ways. Our study specifically examines mitochondrial function, particularly substrate-specific oxidative phosphorylation (OXPHOS) pathway engagement and respiratory fluxes measured in permeabilized tissues. To improve clarity, we have replaced the term "mitochondrial metabolism" with "mitochondrial function" where needed in the manuscript. We also added a conclusive sentence to the paragraph to clarify what is missing here. We believe this revision more accurately reflects the scope of our measurements and addresses the reviewer's concern.
- Comment: "I would not call this a 'rare exception' in the context of mitochondrial metabolism."

- Response: We clarify why the study of Corona et al., is in fact, a rare exception (Lines 93-96). It is the only study considering differences in tissues, and in castes, at different ages. We also replace with wording that accurately situates each referenced study within the broader landscape of honeybee mitochondrial research.

- Comment: “Clarify whether mitochondrial genes are nuclear-encoded or mitochondrial-encoded.”
    - Response: We revised the section (Lines 96-99) to specify which genes analyzed in Corona & Hughes (31) are mitochondrial DNA–encoded (Cyt b, COX-I) and which ones are nuclear-encoded (Cyt c, clock-1, mitochondrial translation initiation factor 2).
- Comment: “Please clarify that you are addressing tissue-specific metabolism (and define ‘metabolism’ earlier).”
    - Response: We now state explicitly that our study focuses on tissue-specific mitochondrial function.
- Comment: “This paragraph suggests you may be unaware of Cervoni et al. (2017).”
    - Response: The reference to the work of Cervoni et al. 2017 was already cited in the paragraph (Lines 104-106), three time in the last sentence. So we have acknowledged their compartment-specific analysis of mitochondrial capacity, in honeybee workers. We explicitly situate their work alongside other studies examining mitochondrial biology in worker tissues. I’m not sure what the reviewer means here?
- Comment: “Throughout the Introduction, the level of analysis you address is unclear.”
    - Response: In addition to modifying this paragraph, we also reviewed earlier and later portions of the introduction to ensure consistent terminology and to clarify the level of mitochondrial analysis addressed throughout the manuscript.

4) “The objective…” paragraph – is it necessary to add the term “cross-sectional” (the meaning is not immediately clear) or could you just state that you are comparing queens and workers?; by “aging” I assume you mean comparing early and late in life, by “task specialization” I assume you mean queen versus worker, but by “longevity” I assume you mean, within each of these groups, individuals or genetic strains that show lifespan diLerences. Is this what you mean?; the “head” is not a tissue, as you clarify when you describe its contents (at least the brain). Please make sure you do not refer to the head as a tissue. Also, the glands of the head are very metabolically active, and diLerentially so for castes and worker specializations. I would assume they will contribute a lot to metabolic variation; “The functional measurement…” I think you mean diLerent pathways that are linked to oxidative phosphorylation, as I think most would consider oxidative phosphorylation a pathway in and of itself; throughout this paragraph I do not see a clear justification for why you are focusing on this component of mitochondrial function. I think if you reduce the generalities in the earlier parts of the Introduction, this will be easier to pull together.

A fully revised paragraph (Lines 110-135) that corrects terminology, clarifies concepts, and responds to all reviewer concerns. Here is the point-by-point response:

- Comments: “Is it necessary to add the term ‘cross-sectional’… or could you just state that you are comparing queens and workers?” “By ‘aging’ I assume you mean early vs. late life; by ‘task specialization’ queen vs. worker; by ‘longevity’ lifespan differences? Is this what you mean?”
    - Response: We removed the term cross-sectional for clarity. The revised paragraph simply states that we compared queens vs. workers and early vs. late adult life stages. To avoid confusion, we removed the term aging, task specialisation, and longevity in this sentence (Lines 110-113).
- Comment: “The ‘head’ is not a tissue… please do not refer to it as a tissue. The glands are metabolically active.”
    - Response: We corrected this by no longer referring to the “head” as a tissue. Instead, used the term body region or body part (Lines 112-114).
- Comment: “‘The functional measurement…’ I think you mean different pathways linked to oxidative phosphorylation.”

    - Response: Revised accordingly. We now describe these measurements as distinct mitochondrial pathways feeding electrons into the ETS rather than calling them “OXPHOS pathways” (Lines 122-128).
- Comment: “There is no clear justification for focusing on this component of mitochondrial function.”
    - Response: We added explicit justification by these pathways representing the main metabolic entry points into the ETS in honeybees with references added. We also clarify the sentence about the markers of mitochondrial content (Lines 123-131).
- Comment: “If you reduce generalities earlier, this paragraph will be easier to pull together.”
    - Response: We revised earlier sections to use more precise terminology, improving consistency and making this objective paragraph clearer and more cohesive.

Methods:
1) 2.1 – the methods are not detailed enough here to make clear the relatedness, origin, and management of your various treatment groups. Also, there are no citations here in this paragraph even though these methods are extensively used by a lot of groups; Were young queens mated or unmated, or variably so? Did you keep track of mating flights?; I think you mean “plastic tubs”, not “tubes”; You say that “Late-life queens…the same origin colony as workers and early-life queens.” This sounds like you are basically using sisters for all individuals in your treatment groups (queens and workers), but the details here are scant. For example, did you generate 1-day-old queens simply from emerging brood frames? I would assume you did something else to generate these queens from the source colonies. Please differentiate the source colony (which is important for relatedness and genotype) from the experimental colony, which sounds like it was simply used to house individuals once marked. How many source and experimental hives did you use? Stress levels and worker and queen physiology can change substantially across the season. What was the timeframe for the experiment exactly, and did you assess whether the date of introduction to the experimental colony influenced your results? Late-life queens were 109 weeks old, so those were not generated at the same time as the early queens and workers, correct? How were these queens managed exactly over this long timeframe? Sounds like they came from distinct colonies? N=4 is a small number. I know it is hard to track queens this long, and this is probably part of the low sample size.

We provided a clarified, and reviewer-responsive version of the paragraph (Lines 141-170). Here is also a point-by-point response to the reviewer.

- Comment: The methods are not detailed enough regarding relatedness, origin, colony management, queen mating status, source vs. experimental colonies, timeframe, and sample sizes. Please clarify how queens and workers were generated and housed, whether young queens were mated, how long-lived queens were managed, and whether introduction date or colony origin could influence results.
    - Response: We have substantially revised Section 2.1 to clarify the origin, relatedness, and management of all treatment groups. Specifically, we now (i) distinguish clearly between source colonies (used to generate brood and emerging bees) and experimental colonies (used to house marked individuals), (ii) report the number of colonies used in each category, and (iii) describe brood pooling procedures implemented to reduce colony-souce effects.
- Early-life queens were mated. Their origin and handling (emergency queen rearing in queenless nucleus colonies) are now described in detail. Workers were generated from synchronized brood frames from four source colonies and introduced into four healthy queenright colonies; we now cite standard methods accordingly.
- Regarding late-life queens, we have expanded the description of their origin and long-term management. These queens were commercially reared in 2022, maintained for approximately two years in stable colonies at the University of Lethbridge, and originated from the same commercial lineage as the worker and early-queen stock. We now explicitly state that their limited availability accounts for the small sample size (n = 4).

- Finally, the timeframe of all collections (June–August 2024), the use of distinct paint colors to track cohorts, and the randomization of sampling across the four experimental colonies have been added. These details address the potential influence of seasonal or colony-specific variation.
- We believe these additions greatly strengthen the clarity and reproducibility of the methods.

2) “Immediately after homogenate preparation…” – what does this timeframe look like, and what is the approximate range of time from anesthetization to analysis for various individuals and tissues? Mitochondrial function varies substantially as a function of the tissue preparation and extraction protocols. Were all individuals assessed on the same day and in the same oroboros run? How were the samples handled to control for effects of time and handling?

We have added these details to Section 2.2 (Lines 193-211) to clarify the consistency of timing and handling across samples.

We have clarified the exact timing in the revised methods. In brief, the term “immediately” refers to a delay of only a few minutes between homogenate preparation and the start of the OROBOROS assay. We processed bees in small batches and prepared the homogenates only when a chamber was already calibrated and ready to receive the sample. This ensured that the homogenate was introduced into the respirometer within minutes of tissue preparation.

The workflow was as follows: we ran six chambers in parallel (three O2k instruments), and tissues were dissected and homogenized one individual at a time, but only when the next chamber became available. For each bee and each tissue, the interval from anesthetization to the start of respiration measurements was typically under 20 minutes. This approach minimizes variability in mitochondrial function due to preparation delay, as recommended by standard high-resolution respirometry practices. Sample handling and timing were therefore highly standardized across individuals, and variation due to time or tissue degradation was minimized.

3) It seems a bit problematic to compare across “tissues” on the basis of mass, since you weighed full body compartments (the head, the thorax, the abdomen with the gut removed). There is going to be a lot of variation in cuticular mass that is unaccounted for. Did you use a protein assay, e.g., to quantify total protein? That would be a better base of comparison. At the very least, throughout you should not say “per mg tissue” – you are not assessing single tissues (especially in the head), and there is the issue of the cuticle as well.

We agree that comparing fluxes across whole body compartments using mass-based normalization has inherent limitations due to differences in cuticular content and the fact that the head and abdomen are not single, uniform tissues. For this reason, we did not rely on mass-specific flux alone to interpret mitochondrial capacity.

As detailed in our response to the Overview of Concerns (point 1) and in the revised Methods section, our primary analytical approach is based on flux control ratios (FCRs). FCRs internally normalize respiration values to a defined maximal reference state, allowing direct comparison of mitochondrial functional capacity independent of: total tissue mass, cuticular proportion, non-mitochondrial protein content, or differences in mitochondrial density.

This makes FCRs particularly appropriate for tissues containing variable amounts of cuticle, which contains protein but no mitochondria. Expressing flux per total protein content would therefore introduce an additional bias because protein assays do not distinguish mitochondrial from non-mitochondrial protein (including cuticular protein). In contrast, FCRs normalize respiration to the mitochondrial system itself, making them a more accurate proxy for the intrinsic functional capacity of the mitochondria present in each sample.

We do report mass-specific flux values to assess the content of mitochondria in the sample (with CS activity and complex IV activity), but all interpretations about the mitochondrial capacity of the various pathways are based primarily on FCRs for the reasons outlined above. We have now clarified this explicitly in the Methods

(by also adding an experimental trace; Fig 1) and Results to ensure readers understand why FCRs, not mass-based fluxes, form the core of our comparisons.
